# Incidence and Risk Factors of Perioperative Mortality in Pediatric ICU Patients

**DOI:** 10.31480/2330-4871/069

**Published:** 2018-04-06

**Authors:** John Aubrey, Hui Zha, Koichi Yuki

**Affiliations:** 1 Department of Anesthesiology, Critical Care and Pain Medicine, Cardiac Anesthesia Division, Boston Children’s Hospital, MA, USA; 2 Tufe University School of Medicine, Boston, MA, USA; 3 Department of Pediatrics, Union Hospital, Tongji Medical College, Huazhong University of Science and Technology, Wuhan, China; 4 Department of Anaesthesia, Harvard Medical School, Boston, MA, USA

**Keywords:** Pediatric, Intensive care unit, Perioperative mortality

## Abstract

**Background:**

There is a limited data of pediatric patients who presented to the intensive care unit (ICU) and undergo procedures under general anesthesia. The primary objective of this study was to evaluate the mortality of this population and assess the risk factors associated with mortality.

**Methods:**

Retrospective study of electronic medical records of pediatric patients who admitted to medical/surgical ICU and underwent produces under general anesthesia during the same ICU admission was performed. Incidence of mortality was obtained and risk factors associated with these mortalities were examined using Univariable logistic regression analysis.

**Results:**

The mortality of pediatric patients who were admitted to the ICU and underwent procedures under general anesthesia was 12.6%, while the mortalities of patients without procedures under general anesthesia and patients who admitted to ICU for postoperative management were 3.5% and 0.4%, respectively. Higher ASA class, emergency cases, higher ventilator support, more inotrope requirement, positive microbe in blood stream, blood transfusion requirement, and general surgery or hematological procedures were highly associated with mortalities. Among them, positive blood stream infection was highest odds ratio (102.00, 95% confidence interval 9.78–1064.09). The profile of patients with positive blood stream infection showed that most of them had underlying immunological/hematological disorders.

**Conclusion:**

In our institution, pediatric patients who admitted to the ICU and underwent procedures under general anesthesia demonstrated the highest mortality among other patients who admitted to ICU. Risk factor analysis demonstrated that patients with positive blood stream infection had highest odds ratio, and were highly associated with immunological/ hematological disorders.

## Introduction

An increasing number of critically ill patients present to surgical and interventional studies under general anesthesia during their intensive care unit (ICU) stay. In general, the incidence of adverse events, such as respiratory failure, infection and cardiovascular embarrassment, is quite high in patients in the ICU [1]. The study conducted in 69 adult/pediatric ICUs by the United States Critical Illness and Injury Trails Group showed that the ICU had the highest mortality rate among any hospital units, with an estimated mortality of average 10.8% [2]. Thus, these patients are usually considered to be at high risk of perioperative events. In the study of a series of elderly patients who admitted to the ICU by Becker, et al. half of them underwent surgery with a mortality rate of 15% [3]. However, there is a paucity of data reported in the pediatric population in a similar setting. Because more and more critically ill patients are undergoing procedures in current medicine, understanding the morbidity and mortality of pediatric patients undergoing procedures during their ICU stays is critical. Identifying higher risk groups among them is also important.

The American Society of Anesthesiologists (ASA) classification currently used is a subjective assessment system of a patient’s overall health categorized into five classes, developed in 1963 [4]. The relationship between higher ASA scoring and perioperative outcomes has been studied, and higher scores are, for the most part, associated with higher rates of complications [5]. Accordingly, ASA scores may quantify patients’ physiological reserve to some extent. However, the ASA scoring system does not specify the content of unwellness, suggesting a lack of specificity. Critically ill patients in the ICU are, in most cases, categorized to higher ASA score groups, and are likely to have more perioperative complications than general population. Identifying risk factors of perioperative complications among this population will help practitioners to prepare for potential issues, thereby hopefully improving patient outcomes. Here we evaluated the incidence of mortality in patients in pediatric ICU in our institution and assessed the perioperative factors associated with mortality of pediatric patients who were admitted to the ICU and underwent procedures under general anesthesia during the same ICU stay.

## Methods

### Data collection

After the Institutional Review Board (IRB) approval, data were retrospectively collected from the electronic medical record of pediatric patients (less than 18-years-old) who were admitted to the Medical/Surgical ICU between January 2011 and December 2014 in Boston Children’s Hospital and then underwent surgical, interventional, or imaging procedures under general anesthesia during the same ICU stay. Consent was waived by the IRB. Patients with congenital heart disease/acquired cardiac issues are primarily admitted to cardiac ICU in our institution. Because they are already considered to be at higher risk of perioperative events than patients without cardiac diseases [6], we did not include this population in this study. Patients who underwent their procedure(s) immediately prior to ICU admission were excluded because their admissions were often due to postoperative pain control and surgical related issues, not due to their preoperative health status. Patients who were placed on extracorporeal membranous oxygenation (ECMO) during their stay were also excluded from the study because ECMO initiation could affect the ventilator setting, hemodynamic support, laboratory values and mortalities. We identified 79 patients eligible for this study. We collected the following information: Age, gender, primary diagnosis, comorbidities, procedures, medications administered in ICU, blood transfusion, documented infections, ASA classification at the time of procedures, ventilatory support, and laboratory values. We did not collect information after patients were transferred to the regular floor. Missing data were left as blank.

### Statistical analysis

Categorical data were expressed as number and percentage, and continuous variables were expressed as median and interquartile range. Normality was measured using the Shapiro-Wilk test. Univariable comparisons of various parameters were performed using Wilcoxon rank test or logistic regression to separately examine the relationship between patient characteristics and mortalities (or positive blood stream infection). The results were expressed as the odds ratio (OR) as a measure of risk, the 95% confidence interval (CI), and P values obtained from the Wald test. The statistical analyses were performed in Stata 13 (College Station, Texas, USA). *P < 0.05* was considered as statistically significant.

## Results

### The outcomes of patients who admitted to ICU

We have identified 79 patients who were aged less than 18 years and underwent procedures under general anesthesia during the same ICU admission between 2011 and 2014 (Table 1). The mortality of this population was 12.6% during the ICU admission. In contrast, the mortality of patients who admitted to ICU and did not have surgery during the same period was 3.5%. Admission to ICU for postoperative care was the most common indication for ICU admission with the lowest mortality of 0.4%. These data suggested that patients who underwent procedures under anesthesia during ICU admission were associated with highest mortality and posed significant challenges to caregivers.

### Patient characteristics, procedures, and complications

Characteristics of 79 patients are summarized in Table 2. Their median age was 7.0-years-old, and they were categorized into either ASA III or ASA IV. 25% of patients were electively admitted to ICU due to their need of respiratory support. The rest of patients were non-electively admitted to ICU for their urgent medical need.

### Univariable analysis of risk factors associated with mortalities

First, we analyzed the factors that were associated with mortalities in the ICU. The factors associated with mortalities in the ICU were as follows; higher ASA class, emergency cases, higher mechanical ventilator support (higher mean airway pressure, high frequency oscillatory ventilation (HFOV) use), hemodynamic support (higher number of inotropic support, use of dopamine, epinephrine and norepinephrine), inhaled nitric oxide use, positive bloodstream infection, blood transfusion (red blood cell (RBC) transfusion, platelet transfusion, fresh frozen plasma (FFP) transfusion), higher number of sedatives, use of midazolam and morphine, higher number of antibiotics, use of antifungal and antiviral drugs, higher white blood cell (WBC) counts, higher lactate, and procedures involving general surgery and hematology (Table 3). Among them, ASA IV (O.R. 23.68), emergency cases (O.R. 22.00), HFOV (O.R. 29.14), epinephrine use (O.R. 88.00), positive bloodstream infection (O.R.102.00), blood transfusion (RBC 26.67, platelet 68.00, FFP 102.00), hematology procedures (O.R. 29.14) showed odds ratio of > 20.

### Patients’ characteristics of positive bloodstream infection

Positive blood stream infection showed the highest O.R. for mortalities in our patients (Table 3). This is in agreement with the literature that bacteremia poses a significant risk to the outcome of adult patients in ICUs [7]. A summary of patients with positive blood stream infection is listed in Table 4. All the patients who died in ICU with blood stream infection were positive for virus or fungus. Also six out of 7 patients had underlying immunological or hematological diseases. Thus, we analyzed the underlying co-morbidities for these patients. When preexisting medical issues exist, we divided them into 1) Hematological/Immunological, 2) Hepatic/Renal, 3) Gastrointestinal, 4) Neurological/Muscular, 5) Respiratory, 6) Vascular and 7) Metabolic/Chromosomal abnormality. As shown in Table 5, preexisting hematological/ immunological illness was significantly associated with positive blood stream infection.

## Discussion

Here we studied the mortality of pediatric patients who were admitted to ICU and underwent procedures under general anesthesia, and also assessed risk factors associated with mortalities. Although we intuitively acknowledge these patients as high-risk for complications, their mortality was in fact extremely high (12.6%). Some of the risk factors associated with mortality in this cohort were intuitive and included the presence of significant respiratory, cardiovascular supports and active blood stream infection, with active bloodstream infection being the highest odds ratio. Interestingly the majority of patients with positive blood microbes had viremia and/or fungemia, and also had hematological and/or immunological existing illness.

As ASA classification indicated in our patients, ASA classification did not differentiate high-risk patients in detail. Preexisting hematological/immunological disease and positive blood stream infection were associated with higher risk in our cohort. If patients on high respiratory, cardiovascular support as well as antibiotic coverage, further attention is required particularly in this population. Because blood stream infection was a significant factor in mortality in our series, it may be important to undertake universal precautions to minimize the further risk of infection. Well established interventions to mitigate the chance of additional postoperative infection include antibiotic prophylaxis, hand hygiene, aseptic techniques during invasive procedures, and perioperative thermoregulation [8]. In addition, the use of higher inspired oxygen and good glycemic control is considered to have additive value. Certainly, these precautions will be particularly important for immunocompromised patients.

The data was gathered from a single center with a sample size considerably smaller than obtainable from a large multi-institutional database. Thus, whether our findings are representative of the experience of other institutions remains to be determined and the non-generalizability of our findings represent the major limitation of this study. In summary, this study has identified that pediatric patients who admitted and underwent procedures during the ICU stay showed a significantly high mortality, and positive bloodstream infection was highly associated with mortalities.

## Figures and Tables

**Table 1:** Mortalities of pediatric patients who admitted to ICU.

Characteristics of patients	Total number	Mortality
Age = < 18 yo, no ECMO run, no surgery during or immediately before ICU admission	1127	39 (3.5%)
Age = < 18 yo, admission postoperatively	4021	16 (0.4%)
Age = <18 yo, no ECMO run, surgery during ICU admission	79	10 (12.6%)

Yo: year old; ECMO: extracorporeal membrane oxygenation.

**Table 2:** Patients’ demographics.

**Age**	7.0 [1.0, 13.0] (years)
**Weight**	20.0 [9.6, 36.4] (kg)
**Sex**	Male 48/Female 31
**ASA class**	
**ASA III**	51/79 (64.6%)
**ASA IV**	28/79 (35.4%)
**Non-elective admission**	59/79 (74.7%)
**Non-survivor**	10/79 (12.7%)
**Duration of ICU stay**	8.0 [4.0, 22.0] (days)
**Number of procedures**	104 (cases)

Age, weight and duration of ICU stay were shown as median [25%, 75% percentile].

**Table 3:** Univariable analysis of factors associated with mortalities.

	Survivor	Non-survivor	O.R. (95% C.I.)	p value
**Age**	8.0 [1.0, 13.0] (years)	3.0 [0.7, 11.5] (years)	0.95 (0.85–1.07)	0.430
**ASA class**				
**ASA III**	50/69 (72.5%)	1/10 (10.0%)	Reference	
**ASA IV**	19/69 (27.5%)	9/10 (90.0%)	23.68 (2.81–199.79)	0.004
**Emergent cases**	3/69 (4.3%)	5/10 (50.0%)	22.00 (4.03–119.91)	<0.0001
**Admission reason**				
**Respiratory cause**	56/69 (81.2%)	8/10 (80.0%)	0.71 (0.07–6.92)	0.930
**Neurological cause**	8/69 (11.6%)	1/10 (10.0%)	0.62 (0.03–12.41)	0.882
**Others**	5/69 (7.2%)	1/10 (10.0%)	Reference	
**Duration of ICU stay**	8.0 [4.0, 21.5] (days)	10.5 [3.0, 24.8] (days)	1.02 (0.96–1.08)	0.565
**Vent dependency**	30/69 (43.5%)	2/10 (20.0%)	0.33 (0.06–1.64)	0.174
**Tracheostomy**	25/69 (36.25)	1/10 (10.0%)	0.13 (0.02–1.63)	0.132
**History of prematurity**	10/69 (14.5%)	1/10 (10.0%)	0.66 (0.07–5.75)	0.703
**Intubated at admission**	49/69 (71.0%)	5/10 (50.0%)	0.41 (0.11–1.57)	0.191
**Preoperative elective admission**	20/69 (29.0%)	0/10 (0%)	n/a	n/a
**Intubated during ICU stay**	55/69 (79.7%)	10/10 (100%)	n/a	n/a
**NIPPV use**	15/69 (21.7%)	4/10 (40.0%)	2.4 (0.60–9.62)	0.217
**Highest mean airway pressure**	13.0 [11.0, 16.0] (cmH_2_O)	17.5 [15.0, 30.5] (cmH_2_O)	1.29 (1.07–1.55)	0.009
**Lowest SpO_2_/FiO_2_**	183.0 [93.8, 267.8]	73.7 [32.0, 88.3]	0.97 (0.94–0.99)	0.011
**Highest SpO_2_/FiO_2_**	323.0 [268.0, 447.0]	242.5 [92.3, 273.3]	0.98 (0.97–0.99)	0.002
**HFOV**	1/69 (1.4%)	3/10 (30.0%)	29.14 (2.66–319.06)	0.006
**Steroid use**	37/69 (53.6%)	7/10 (70.0%)	2.02 (0.48–8.46)	0.337
**Number of inotrope**	0 [0, 0]	3 [2, 4]	9.40 (2.76–31.98)	<0.0001
**Dopamine**	13/69 (18.8%)	7/10 (70.0%)	10.05 (2.29–44.20)	0.002
**Epinephrine**	3/69 (4.3%)	8/10 (80.0%)	88.00 (12.72–608.59)	<0.0001
**Norepinephrine**	2/69 (2.9%)	9/10 (90.0%)	301.5 (24.77–3670.34)	<0.0001
**Vasopressin**	0/69 (0%)	4/10 (40.0%)	n/a	n/a
**Milrinone**	1/69 (1.4%)	1/10 (10.0%)	7.56 (0.43–131.62)	0.165
**iNO use**	3/69 (4.3%)	3/10 (30.0%)	9.43 (1.59–55.90)	0.013
**Reintubation**	4/69 (5.8%)	2/10 (20.0%)	4.06 (0.64–25.82)	0.137
**Documented infection**	38/69 (55.1%)	8/10 (80.0%)	3.26 (0.65–16.50)	0.153
**Blood culture**	1/69 (1.4%)	6/10 (60.0%)	102.00 (9.78–1064.09)	<0.0001
**Urine culture**	3/69 (4.3%)	1/10 (10.0%)	2.44 (0.23–26.09)	0.459
**Sputum/tracheal culture**	27/69 (39.1%)	4/10 (40.0%)	1.04 (0.27–4.02)	0.958
**Would culture**	6/69 (8.7%)	2/10 (20.0%)	2.58 (0.44–15.04)	0.291
**Transfusion**				
**RBC transfusion**	9/69 (13.0%)	8/10 (80.0%)	26.67 (4.87–146.05)	<0.0001
**Platelet transfusion**	1/69 (1.4%)	5/10 (50.0%)	68.00 (6.61–699.75)	<0.0001
**FFP transfusion**	1/69 (1.4%)	6/10 (60.0%)	102.00 (9.78–1064.09)	<0.0001
**Number of sedatives**	1.0 [0.0, 3.0]	2.5 [1.8, 4.3]	1.54 (1.05–2.25)	0.026
**Midazolam**	26/69 (37.7%)	8/10 (80.0%)	6.62 (1.30–33.57)	0.023
**Morphine**	19/69 (27.5%)	6/10 (60.0%)	3.95 (1.00–15.55)	0.050
**Fentanyl**	12/69 (17.4%)	4/10 (40.0%)	3.17 (0.77–12.97)	0.109
**Propofol**	13/69 (18.8%)	2/10 (20.0%)	1.08 (0.20–59.68)	0.930
**Dexmedetomidine**	14/69 (20.3%)	4/10 (40.0%)	2.62 (0.65–10.56)	0.176
**Methadone**	9/69 (13.05)	2/10 (20.0%)	1.67 (0.30–9.13)	0.556
**Ketamine**	1/69 (1.4%)	1/10 (10.0%)	7.56 (0.43–131.62)	0.165
**Phenobarbital**	4/69 (5.8%)	1/10 (10.0%)	1.81 (0.18–18.00)	0.615
**Number of antibiotics**	1.0 [1.0, 1.0]	2.0 [1.0, 2.0]	1.33 (1.05–1.68)	0.017
**Antifungal**	8/69 (11.6%)	6/10 (60.0%)	11.44 (2.65–49.45)	0.001
**Antiviral**	7/69 (10.1%)	1/10 (10.0%)	0.98 (0.11–8.96)	0.989
**Lowest WBC count**	7.5 [5.0, 9.3]	6.9 [2.2, 9.9]	0.92 (0.76–1.12)	0.410
**Highest WBC count**	13.4 [9.2, 18.8]	23.0 [15.0, 40.2]	1.06 (1.01–1.12)	0.027
**Lowest platelet count**	232.0 [171.8, 298.8]	35.5 [10.5, 84.0]	0.98 (0.96–0.99)	<0.0001
**Highest lactate**	1.4 [1.0, 2.0]	5.2 [2.5, 14.8]	6.22 (1.58–24.55)	0.009
**Highest creatinine**	0.3 [0.2, 0.6]	1.2 [0.7, 2.1]	1.55 (0.87–2.70)	0.137
**Procedures**				
**ORL**	30/69 (43.5%)	2/10 (20.0%)	0.37 (0.08–1.77)	0.213
**Radiology**	11/69 (15.9%)	2/10 (20.0%)	1.32 (0.25–7.06)	0.747
**Orthopedics**	6/69 (8.7%)	0/10 (0%)	n/a	n/a
**Gastroenterology**	10/69 (14.5%)	0/10 (0%)	n/a	n/a
**General surgery**	10/69 (14.5%)	5/10 (50.0%)	5.90 (1.44–24.15)	0.014
**Urology**	3/69 (4.3%)	0/10 (0%)	n/a	n/a
**Dental**	0/69 (0%)	0/10 (0%)	n/a	n/a
**Plastics**	1/69 (1.4%)	0/10 (0%)	n/a	n/a
**Dermatology**	3/69 (4.3%)	0/10 (0%)	n/a	n/a
**Neurosurgery**	10/69 (14.5%)	0/10 (0%)	n/a	n/a
**Ophthalmology**	1/69 (1.4%)	0/10 (0%)	n/a	n/a
**Hematology**	1/69 (1.4%)	3/10 (30.0%)	29.14 (2.66–319.06)	0.006
**Pulmonary**	8/69 (11.6%)	1/10 (10.0%)	0.85 (0.09–7.60)	0.882
**Maxillofacial**	1/69 (1.4%)	0/10 (0%)	n/a	n/a

Data were shown as median [25, 75 Percentiles] or number (percentage). O.R: odds ratio; CI: confidence interval; ICU: intensive care unit; Vent: ventilator; NIPPV: non-invasive positive pressure ventilation; SpO_2_/FiO_2_: oxygen saturation%/inspired oxygen concentration ratio; HFOV: high frequency oscillatory ventilation; iNO: inhaled nitric oxide; RBC: red blood cell; FFP: fresh frozen plasma; WBC: white blood cell; ORL: otorhinolaryngology.

**Table 4:** Characteristics of patients with positive blood stream infection.

Age (years)	Admission diagnosis	Underlying disease	Blood culture/PCR results	ICU mortalities
13	Respiratory distress	Autoimmune hepatitis	EBV	yes
5	Respiratory distress	Hemophagocytic lymphohistiocytosis	*Aspergillus galactomannan*	yes
10	Respiratory distress	AML	*Aspergillus galactomannan*, EBV, *adenovirus*	yes
14	Respiratory distress	AML	*Enterococcus faecalis*	no
0.42	Respiratory distress	Autoimmune lymphoproliferative syndrome	*Adenovirus*	yes
1	Respiratory distress	Hemophagocytic lymphohistiocytosis	*Aspergillus galactomannan*, EBV	yes
0.75	Respiratory distress	Biliary atresia	*Candida albicans*	yes

AML: acute myeloid leukemia; EBV: Epstein Barr virus.

**Table 5:** Analysis of positive blood stream infection versus co-morbidities.

	O.R. (95% CI)	p value
**Hematological/Immunological**	48.00 (5.11–451.31)	0.001
**Hepatic/renal**	1.17 (0.13–10.84)	0.892
**Gastrointestinal**	n/a	n/a
**Neurological/muscular**	n/a	n/a
**Respiratory**	n/a	n/a
**Vascular**	n/a	n/a
**Metabolic/Chromosome abnormality**	n/a	n/a

O.R: odds ratio; CI: confidence interval.
